# *In-vitro* Bioactivity and Phytochemical Screening of Extracts from Rhizomes of *Eremostachys azerbaijanica* rech. f. Growing in Iran

**Published:** 2017

**Authors:** Solmaz Asnaashari, Abbas Delazar, Parina Asgharian, Farzaneh Lotfipour, Sedigeh Bamdad Moghaddam, Fariba Heshmati Afshar

**Affiliations:** a *Biotechnology Research Center, Tabriz University of Medical Sciences, Tabriz, Iran. *; b *Drug Applied Research Center, Tabriz University of Medical Sciences, Tabriz, Iran. *; c *Faculty of Pharmacy, Tabriz University of Medical Sciences, Tabriz, Iran. *; d *Student Research Committee, Tabriz University of Medical Sciences, Tabriz, Iran. *; e *Faculty of Traditional Medicine, Tabriz University of Medical Sciences, Tabriz, Iran.*

**Keywords:** *Eremostachys azerbaijanica*, general toxicity, cytotoxicity, antimicrobial, antioxidant

## Abstract

The current study evaluated the general toxicity, antioxidant, antimicrobial, and cytotoxic activity of extracts obtained from the rhizomes of *Eremostachys azerbaijanica* (Labiatae) as well as analyzed the potent extracts using GC-MS. Extracts of *E. azerbaijanica* in n-hexane, dichloromethane (DCM) and methanol (MeOH) were prepared using a Soxhlet apparatus. The antioxidant activity of the extracts was evaluated for free radical scavenging activity by DPPH assay. The antimicrobial activity of samples was determined by disc diffusion and brine shrimp lethality assay (BSLA) was used to assess general toxicity. The cytotoxicity of each extract was determined by MTT assay against human colorectal adenocarcinoma (HT29), human lung carcinoma (A549) and a normal cell line (human umbilical vein endothelial cells, HUVEC).

The MeOH extract showed significant antioxidant activity and the n-hexane and DCM extracts showed promising activity against gram-positive species when compared with amikacin as a standard. Moreover, the n-hexane extract displayed the most potent activity in general toxicity assay. The results showed that all three extracts have cytotoxic effects against the A549 cell line. In the case of HT29 cell lines, only the DCM extract exhibited cytotoxicity. Interestingly, none of the extracts showed significant cytotoxic activity against the HUVEC cell line. The bioassay-guided identification of constituents showed the presence of fatty acids and steroids as the compounds responsible for bioactivity in the non-polar extracts.

## Introduction

The discovery of effective drugs derived from plants is of major interest to scientists for treatment of infections and cancers (1). Numerous sources of lead molecules from different species of plants have been introduced. These chemotherapeutic compounds include taxol from *Taxus brevifolia *L., camptothecin from *Camptotheca acuminate*, vinca alkaloids from *Catharanthus roseus*, and podophyllotoxin from *Podophyllum peltatum *L. (2, 3). The increase in resistance to routine antibiotics makes discovery of new sources of antimicrobial compounds an urgent necessity (4). The systematic screening of plant extracts for their anti-cancer and antibacterial have been started to find new natural products with potential activity against malignant cells and multi-resistance bacteria. In this regard, our study focused on an Iranian plant, *Eremostachys azerbaijanica *rech. f. from the genus* Eremostachys *(family: Lamiaceae alt. Labiatae; subfamily: Lamioideae), which are distributed throughout central and western Asia. This is a large genus with approximately 60 to 80 species that is generally restricted to the dry mountains of the flora Iranica area from southeastern Turkey and western Iran to central Asia and Afghanistan (5, 6). It is represented by 15 endemic species in Iranian flora with its common Persian name of *sonbol-e-biabani*. These plants includ perennial herbs with robust stems, thick roots, laciniate leaves, large calyces, and large creamy corollas bearded nutlets (5)-(8). Plants of this genus are known as important medicinal herbs and have been used in indigenous or folk medicine. The roots and rhizomes of *Eremostachys glabra* (*E. glabra* Boiss) is traditionally used as a topical analgesic and anti-inflammatory agent in Iran (5). Moreover, decoction of the roots and flowers of* E. laciniata *has been applied to treat allergies, headache, and liver disease (9, 10). Previous studies have revealed that *E. laciniata* is a potential source of natural components with analgesic (9), anti-depressant (11), antinociceptive (12), and anti-inflammatory (9, 13) properties. Furthermore, preceding investigations have reported the antioxidant and antibacterial effects of these plants (14-16). 

Phytochemical studies on different extracts of *Eremostachys* genus have revealed the presence of several flavonoids (e.g., luteolin, chrysoeriol glycosides), monoterpene glycosides, ferulic acid derivatives (6), phenylethanoids, phytosterols (9), and iridoid glucosides (14, 17). Examination of the essential oil composition of the plants of this genus has shown that they are rich in monoterpenes and sesquiterpenes (18-20). The current study evaluated the rhizomes of *E. azerbaijanica* rech. f. for its antioxidant and antimicrobial properties along with its general toxicity and cytotoxic activities. The active extracts were further subjected to gas chromatography mass spectrometry (GC-MS) for identification of the active compounds present in the extract.

## Material and Methods

*Plant Material*

The rhizomes of *E. azerbaijanica *Rech.f. were collected during July 2012 from Sahand mountains in East Azarbaijan province (Northwest Iran), 1850 m above sea level, GPS coordinates: N 37°45› 32.4» E 45° 58› 41.9» . A voucher specimen has been retained in the herbarium of the Faculty of Pharmacy, Tabriz University of Medical Sciences, Iran under the accession code TBZ-fph-738.

*Extraction*

Air-dried and ground rhizomes of *E. azerbaijanica *(100 g) were Soxhlet extracted respectively with n-hexane, DCM and MeOH (1.1 L each). All these extracts were separately concentrated using a rotary evaporator at a maximum temperature of 45 °C.

*Brine Shrimp Lethality Test (BSLT)*

The general toxicity of different extracts of* E. azerbaijanica *rhizomes were monitored by the BSLT method. The *Artemia salina* eggs were hatched in a conical shaped vessel containing 300 mL artificial sea water prepared from commercial sea salt (40g/L). The flasks were well aerated with the aid of an air pump, and kept in a water bath at 29-30 ^o^C. A bright light source was left on. After 48 h, active nauplii were collected from the bright compartment of hatching tank and used for the assay. The extracts were dissolved in dimethylsulfoxide (DMSO) and diluted with artificial sea water so that final concentration of DMSO did not exceed 0.05 percent. Different concentrations of extract were prepared by serial dilution from stock sample (1mg/mL). 1 mL of each concentration along with 10 mL of aerated sea water was transferred into clean sterile universal vials. About 10 nauplii were introduced into each vial and incubated for 24 h. The controls were DMSO (negative control) and podophyllotoxin (positive control). Finally, the number of survivals at each dosage of extracts and controls were counted and recorded. The lethal concentration of each extract resulting in 50 percent mortality of the brine shrimp (LC_50_) was calculated using linear regression analysis by Excel software (6).

*Free Radical Scavenging Activity Test (FRST)*

Antioxidant activity of the extracts was assessed spectrophotometrically using the 2, 2-diphenyl-1-picrylhydrazyl (DPPH) radical (molecular formula C_18_H_12_N_5_O_6_, molecular weight 394) obtained from Sigma Aldrich Company. Stock solutions of extracts were prepared as 1 mg/mL in chloroform (CHCl_3_) for n-hexane and DCM extracts and MeOH for MeOH extract. 

Serial dilutions were made to obtain concentrations of 5×10^-1^, 2.5×10^-1^, 1.25×10^-1^, 6.25×10^-2^, 3.13×10^-2^ and 1.56×10-^2 ^mg/mL. Diluted solutions of extracts (5 mL each) were mixed with 0.08 mg/mL DPPH solution (5 mL) and allowed to stand for 30 min for occurring any reaction. The UV absorbance was recorded at 517 nm. The experiment was done in triplicate and the reduction of free radical DPPH in percent (R %) was calculated in the following equation:

R% = (A _Negative control_ – A _sapmle_)/ A _Negative control_) × 100

Where A _Negative control_ is the absorbance of the negative control (containing all the reagents except the extract), and A _sapmle_ is the absorbance of the test samples. Extract concentration providing 50% reduction (RC_50_) was calculated from the graph plotting reduction percentage against extract concentration. Quercetine was used as positive control (6).

*Cytotoxicity Assay*

HT29 (human colorectal adenocarcinoma), A549 (human lung carcinoma) and HUVEC (Human Umbilical Vein Endothelial) cell lines were cultured in *RPMI 1640* (*Roswell Park Memorial Institute*) medium with suitable additives containing 100 IU/mL penicillin and 100 μg/mL streptomycin supplemented with 10% fetal bovine serum (FBS). The cells were cultured in a humidified atmosphere of a 5% CO_2_ at 37 °C. For MTT assay, the cells were seeded at a density of 1 ×10^4^ cells/well into 96-well plates and incubated for 24 h before the cells were exposed to different concentration of extracts (including 1, 10, 100, 1000 µg/mL) and incubated for 3 days in a humidified atmosphere at 37 °C in presence of 5% CO_2_. After 72 h of incubation each well received 20 µL of 3-(4, 5-dimethylthiazol-2-yl)-2, 5-diphenyltetrazolium bromide reagent (MTT; 5 mg/mL in phosphate- buffered saline), and the plates were incubated at 37 °C for 4 h. The amount of MTT reduction was quantified spectrophotometrically at 570 nm using a microplate reader (ELISA plate reader, Bio teck, Bad Friedrichshall, Germany). The experiments performed in triplicate and for comparing the anti proliferative activity of plants, Paclitaxel and DMSO were considered as positive and negative controls, respectively. 

The cell survival was calculated by the following formula:

Relative viability (%) = (A _test_/A _control_) ×100

Where A_control_ is the absorbance of the negative control and A_test_ is the absorbance of the sample. The cytotoxicity was expressed as *IC*_50_ values (the concentration of extract inhibiting the cell growth by 50%) which determined with the Sigma Plot 10 software (21- 23).

*Antimicrobial Assay*

*Microbial Strains*

Microorganisms were obtained as lyophilized culture from Persian Type Culture Collection (Iran). Organisms were as follows: two species of Gram negative bacteria, *Pseudomonas aeroghinosa* (ATCC 9027), *Escherichia coli* (ATCC 8739), and two strains of Gram positive species, *Staphylococcus epidermidis *(ATCC 12228), *Staphylococcus aureus* (ATCC 6538) and one species of moulds, *Candida albicans* (ATCC 10231).

**Table 1.** BSLT, FRST and cytotoxicity of n.hexane, dichloromethane and methanol extracts of *E. azerbaijanica* rhizomes.

**EAR extracts**	**General toxicity** * **(g/mL** ** )**	**Antioxidant** ** **(g/mL** ** )**	**A549** **(g/mL)**	**Cytotoxicity** **HT29(g/mL)**	**HUVEC** **(g/mL** ** )**
n.hexane	63± 1.1	989± 31	160.82±54.81	1000	1000
DCM	881± 6	747.2± 19.1	400.23±50.01	116.07± 30.14	1000
MeOH	1000	264.1± 9.7	604.64±82.41	1000	1000

The Experiment was performed in triplicate and expressed as Mean ± SD.

* The LC_50_ value of podophyllotoxin as positive control was 2.8 ± 0.1g/mL.

** The RC_50_ value for quercetin as positive control was 3.9 ± 0.1 g/mL

**Table 2:** Volatile constituents of the n.hexane and DCM extracts of *E. azerbaijanica* rhizomes

**Extracts **	**Total identified content (%) **	**Compounds (content %)**
n.hexane	93.64	Fatty acids and derivatives (61.64%):
		Palmitic acid(8.01%), Palmitic acid ethyl
		ester (0.35%),
		Methyl linolelaidate (0.38%), Linoleic acid
		(50.08%),
		Oleic acid (2.82%)
		Steroids derivatives (14.11%):
		Androlone(0.85%),Clionasterol (1.56%),
		Stigmasterol (1.68%),
		Campesterol (2.02%), Stigmast-4-en-3-one (2.20%),
		beta.-Sitosterol(5.80%).
		Linear alcohol (2.88%):
		Cyclopentanol, 3-methyl-(1.22%),
		1-Pentanol, 2, 2-dimethyl-(1.66%).
		Cyclic alcohol (2.52%):
		Thunbergol (2.52%).
		Heterocyclic hydrocarbons (3.04%):
		Armillarisin A (1.85%),
		Tetrahydrofuran, 2, 2-dimethyl-(1.19%)
		Linear ketones (9.45%):
		1-Butenyl methyl ketone (9.45%)
DCM	90.59	Steroids and derivatives (56.85%):
		4, 4- Dimethylandrost-5-en-3-ol (56.85%)
		Heterocyclic hydrocarbons (10.76%):
		N-Aminopyrrolidine(2.35%), Cyclopenta[c]pyran-4-
		Carboxylic acid, 7-methyl-, methyl ester (4.05%),
		Armillarisin A (4.36%)
		Sesquiterpenes (12.18%): Epiglobulol (8.09%),
		2, 6, 10, 10-Tetramethylbicyclo [7.2.0] undeca-2, 6-diene (4.09%).
		Alkanes (10.81%):
		Tetracosane (7.36%) ،Octacosane(3.44%).

**Table 3:** Antibacterial activity of n.hexane, DCM and MeOH extracts from rhizomes of *E. azerbaijanica*.

Bacterial species	Inhibition zone diameter (mm)			
	n.hexane extract	DCM extract	MeOH extract	Amikacin ( positive control)
*Staphylococcus aureus*	15± 0	16 ± 0.14	N/A	22 ± 0.43
*Staphylococcus epidermidis*	13 ± 0.14	1 ± 0	N/A	21 ± 0.21

The disc diameter was 6 mm.

*Disc-Diffusion Assay*

Activated microorganisms were cultivated on *Muller Hinton* Broth medium (Sigma-Aldrich). Cell cultures were incubated overnight at 37 °C. Saline solution was twice applied to provide the turbidity for the centrifuged pallets at 3000 rpm for 15 min (equal to 0.5 Mc Farland, 10 ^8 ^CFU/mL as a standard optical density). The final concentration of inoculums was adjusted to about 10 ^6 ^CFU/mL with sterile saline solution. The dried plant extracts were dissolved in 50% aqueous DMSO to a final concentration of 1mg/mL. The antimicrobial activities of the plant extracts were determined by paper disc diffusion assay (23, 24). To obtain a uniform microbial growth, 10 mL of prepared inoculums suspensions were spread over the autoclaved Muller Hinton Agar plates (Merck). Sterilized filter paper discs (Whatman paper with 6 millimeters diameter) were impregnated with 50 µL of different concentrations of extracts and placed on the surface of the media. The plates were incubated for 30 min in refrigerator to allow the diffusion of extract, and then they were incubated at 37 °C for 24 h. Finally the inhibition zones obtained around sterile discs were measured. 

To comparing the potency of the antimicrobial activity of the extracts, two control groups were considered: 1. aqueous DMSO as a vehicle (negative) control 2. The standard disc of Amikacin was as a positive control. All experiments were performed in triplicate, and mean value was calculated .Compounds that have illustrated significant antibacterial activity, were selected for further evaluating for their minimum inhibitory concentration (MIC). Extracts were prepared via serial dilutions in broth then added to test tubes which were impregnated with volume of the adjusted inoculums. After incubation at 37 ºC for 24 h the MIC was read. The MIC was defined as the lowest concentration of a fraction which was able to completely inhibit the growth of each bacterial strain (25, 26).

*GC-MS Analysis of Potent Fractions*

GC–MS analyses were carried out on a Shimadzu QP-5050A GC–MS system equipped with a DB-1 fused silica column (60 m × 0.25 mm i.d., film thickness 0.25 µM).

For analyzing n-hexane extract, the oven temperature was held at 50 °C for 1 min, then programmed at 4 °C/min to 230 °C and then 1.5 °C/min to 310 °C . In regard to DCM extract, the oven temperature was held at 50 °C for 1 min, and then programmed at 3 °C/min to 310 °C. Other operating conditions were as follows: carrier gas, helium with a flow rate of 1.3 mL/min; injector temperature, 280 °C; at split ratio, 1:10; Mass spectra was taken at 70 eV (ionization energy); scan time, 1 s; Mass range was from *m/z* 30–600 amu.

*Identification of Components*

Identification of the constituents was based on direct comparison of the retention indices and mass spectral data with those for standard alkanes, and computer matching with the NIST 21, NIST 107 and WILEY229 libraries, as well as by comparison of the fragmentation patterns of the mass spectra with those reported in the literature (27).

*Statistical Analysis*

All experiments were done in triplicate measurements and presented as the Mean ± SD. Data were analyzed by Excel 2010 Microsoft.

## Results and discussion

The brine shrimp lethality test (BSLT) is a simple method of screening for assessing toxicity of compounds. The free radical scavenging activity test (FRST) is a bioassay for determination of the antioxidant potential of compounds. Both techniques are easily-mastered, low cost, and utilize small amounts of test material (27, 29).

The BSLT and FRST of the extracts obtained from *E. azerbaijanica* rhizomes were accomplished. As depicted in Table.1 both n-hexane and DCM extracts were efficient in BSLT and the n-hexane extract showed significant effect in comparing with podophyllotoxin (a cytotoxic lignan) as a well-known standard. The DCM extract showed moderate toxicity and MeOH extract showed no significant level of toxicity (LC_50 _ 1.0 mg/mL). The BSLT stipulates that a LC_50_ < 1 mg/mL is considered bioactive for toxicity of plant extracts (30). A previous study of BSLT for the pure iridoid glycosides of *E. laciniata* showed that these compounds had no significant level of toxicity at LC_50_ > 1.0 mg/mL [14]. Earlier data showed a good correlation among BSLT, cytotoxicity, and pesticidal activity as well as human anticancer and antitumor properties (31, 32). 

The free radical scavenging activity of the extracts of *E. azerbaijanica* rhizomes were evaluated by DPPH assay (6). The MeOH extract showed the highest DPPH removal activity with RC_50_ value of 264.1 ± 9.7 µg/mL DCM and n.hexane extracts showed RC_50 _values of 747.2 ± 19.1 and 989 ± 31 µg/mL, respectively. Quercetin as a positive standard showed the highest activity with lowest value of RC_50_ (3.9 ± 0.1 µg/mL). These results indicate the presence of some powerful polar antioxidant substances such as phenols and flavonoides in the MeOH extract. Previous investigations have shown a positive correlation between antioxidant properties and phenolic contents (33, 34). 

The presence of the antioxidant compounds such as phenylethanoid glycosides, flavonoids and iridoid glycosids have been demonstrated by previous phytochemical studies on the *E. glabra* and *E. laciniata* species [6, 15,16, 35]. Therefore, the possibility that the antioxidant activity displayed by MeOH extract of* E. azerbaijanica *rhizomes reported here would be due to the presence of these types of compounds could not be excluded (36, 37).

The cytotoxicity activity (IC_50_) of the extracts of* E. azerbaijanica* rhizomes were evaluated for two cancer cell lines and one normal cell line and the results were shown in Table 1. The DCM extract showed the most potent cytotoxicity effect against HT29 cell line, but neither n-hexane nor MeOH extracts showed significant activity against this cell line. Conversely, all three extracts showed various degrees of cytotoxicity against A549 cell line. Among them, the n-hexane extract showed the most potent activity while the MeOH extract was the weakest one.

Additionally, the all three extracts of *E. azerbaijanica *exhibited no significant effect against HUVEC cell lines; so, based on the remarkable effects on cancerous cell lines, this plant can be considered as a potential natural resource of antitumor agents for future studies. In some previous reports, there is a direct relationship between the toxic activity in the BSLT and antiproliferative effects; hence, it might be suggested that the BSLT is an inexpensive, easily mastered and suitable preliminary assay for predicting cytotoxic activity. (38-40).

The potent cytotoxic extracts were analyzed by GC-MS for identifying the bioactive chemical groups of the extracts and the results were shown in Table 2.

Table 2. shows that the fatty acid derivatives and steroids were the major active constituents with amounts of 61.64% and 14.11%, respectively. It appears that the potent cytotoxic effects of n-hexane extract relates to the presence of these compounds. GCMS identified steroids and derivatives (56.85%), heterocyclic hydrocarbons (10.76%), sesquiterpenes (12.18%) and linear alkanes (10.81%) in the DCM extract. These findings are consistent with data obtained in previous research for the cytotoxic effects of fatty acids (41-45), triterpenoids (46), steroids (47), sesquiterpenes (48-50), flavonoid (51,52), heterocyclic hydrocarbons (53,54), and coumarins (55) on different cancer cell lines. The cytotoxic properties exhibited by n-hexane and DCM extracts could also result from the presence of these compounds. In regard to antimicrobial assessments of the extracts (Table 3), the disc diffusion method was carried out against two gram negative species (*Pseudomonas *aeruginosa and *Escherichia coli*), two gram positive species (*Staphylococcus epidermidis *and *Staphylococcus aureus*), and a fungal species (*Candida albicans*).

The n-hexane and DCM extracts showed varying degrees of antimicrobial activity only against the gram positive strains. DCM extract displayed the most potent antibacterial activity against the gram positive species, remarkably against *S. aureus* with MIC value of 55 mg/mL. On the contrary, the MeOH extract exhibited no inhibitory activity against the mentioned microorganisms. A previous study on the rhizomes of* E*. *laciniata* showed that the pure iridoid glucosides isolated from MeOH extract offered low to moderate antibacterial activity (14), however our results are not in consistent with that results. To the best of our knowledge, this is the first report on the toxicity, antioxidant, antibacterial, and cytotoxic effects of the rhizomes of *E. azerbaijanica*. To sum up, our studies demonstrated the *in-vitro* cytotoxic effects of n.hexane and DCM extracts without any deleterious effects on normal cells; therefore, in our forthcoming studies, we should make attempts on isolation of active and pure ingredients and clarification of anti-neoplastic mechanism of them.

## References

[1] Hemanth Kumar M, Dhiman V, Choudhary R, Chikara A (2014). Anticancer activity of hydro alcoholic extracts from Paris polyphylla rhizomes against human A549 lung cancer cell lines using MTT assay. Inter. Res. J. Pharm..

[2] Zare Shahneh F, Valiyari S, Azadmehr A, Hajiaghaee R, Bandehagh A, Baradaran B (2013). Cytotoxic activities of Ferulago angulata extract on human leukemia and lymphoma cells by induction of apoptosis. J. Med. Plants Res..

[3] Nirmala MJ, Samundeeswari A, Sankar PD (2011). Natural plant resources in anti-cancer therapy-A review. Res Plant Biol..

[4] Jayaraman S, Manoharan MS, IIIanchezian S (2008). In-vitro antimicrobial and antitumor activities of Stevia rebaudiana (Asteraceae) leaf extracts. Trop. J. Pharm. Res..

[5] Salamaki Y, Zare S, Heubl G (2012). The genus phlomoides moench ( Lamiaceae; Lamioideae; Phlomideae) in Iran; an updated synopsis. Iran. J. Bot..

[6] Delazar A, Shoeb M, Kumarasamy Y, Byres M, Nahar L, Modarresi M (2004). Two bioactive ferulic acid derivatives from Eremostachys glabra. DARU J. Pharm. Sci..

[7] Mozaffarian V (1996). A dictionary of Iranian plant names: Latin, English, Persian.

[8] Naghibi F, Mosaddegh M, Mohammadi Motamed M, Ghorbani A (2005). Labiatae family in folk medicine in Iran: from ethnobotany to pharmacology. Iran. J. Pharm. Res..

[9] Delazar A, Sarker SD, Nahar L, Jalali SB, Modaresi M, Hamedeyazdan S (2013). Rhizomes of Eremostachys laciniata: isolation and structure elucidation of chemical constituents and a clinical trial on inflammatory diseases. Adv. Pharm. Bull..

[10] Said O, Khalil K, Fulder S, Azaizeh H (2002). Ethnopharmacological survey of medicinal herbs in Israel, the Golan Heights and the West Bank region. J. Ethnopharm..

[11] Nisar M, Khan S, Dar A, Rehman W, Khan R, Jan I (2013). Antidepressant screening and flavonoids isolation from Eremostachys laciniata (L) Bunge. African J. Biotech..

[12] Khan S, Nisar M, Simjee SU, Rehman W, Khan R, Jan I, Momin DS (2010). Evaluation of micronutrients level and antinociceptive property of Eremostachys laciniata (L) Bunge. African J. Biotech..

[13] Khan S, Nisar M, Rehman W, Khan R, Nasir F (2010). Anti-inflammatory study on crude methanol extract and different fractions of Eremostachys laciniata. Pharm. Biol..

[14] Modaressi M, Delazar A, Nazemiyeh H, Fathi-Azad F, Smith E, Rahman MM (2009). Antibacterial iridoid glucosides from Eremostachys laciniata. Phytother. Res..

[15] Alali FQ, Tawaha K, El-Elimat T, Syouf M, El-Fayad M, Abulaila K (2007). Antioxidant activity and total phenolic content of aqueous and methanolic extracts of Jordanian plants: an ICBG project. Nat. Prod. Res..

[16] Delazar A, Gibbones S, Kumarasamy Y, Nahar L, Shoeb M, Sarker SD (2005). Antioxidant phenylethanoid glycosides from the rhizomes of Eremostachys glabra (Lamiaceae). Biochem. Sys. Ecol..

[17] Ali B, Mehmood R, Mughal UR, Malik A, Safder M, Hussain R (2012). Eremosides A–C, new iridoid glucosides from Eremostachys loasifolia. Helv. Chim. Acta..

[18] Manafi H, Shafaghat A (2010). Chemical composition of essential oil of Eremostachys azerbaijanica Rech f from Iran. J. Essent. Oil Bear PL..

[19] Navaei MN, Mirza M (2006). Chemical composition of the oil of Eremostachys laciniata (L) Bunge from Iran. Flav. Fragr J..

[20] Nori-Shargh D, Kiaei S, Deyhimi F (2007). The volatile constituents analysis of Eremostachys macrophylla Montbr & Auch. from Iran. Nat. Prod. Res..

[21] Tofighi Z, Asgharian P, Goodarzi S, Hadjiakhoondi A, Ostad SN, Yassa N (2014). Potent cytotoxic flavonoids from Iranian Securigera securidaca. Med. Chem. Res..

[22] Levy A, Lewis A (2011). Cassia alata leaf extract induces cytotoxicity in A549 lung cancer cells via a mechanism that is caspase 8 dependent. West Indian Med. J..

[23] Zeidooni L, Rezaei M, Tabar MH (2012). Gamma tocopherol and lovastatin additively induced apoptosis in human colorectal carcinoma cell line (HT29). Jundishapur J. Nat. Pharm. Prod..

[24] Sarkar B, Raihan S, Sultana N, Rahman R, Islam ME, Ahmed S (2012). Cytotoxic, antibacterial and free radical scavenging activity studies of the solvent extracts of aerial stems of Equisetum debile roxib. Int. J. Chem. Sci..

[25] Behmanesh F, Pasha H, Sefidgar SAA, Moghadamnia A, Basirat Z (2015). A comparative study of antifungal activity of Lavender brew, Lavender essential Oil, and Clotrimazole: an in-vitro study. Caspian J. Repord. Med..

[26] Motamedi H, Seyyednejad SM, Bakhtiari A, Vafaei M (2014). Introducing Urtica dioica, a native plant of Khuzestan, as an antibacterial medicinal plant. Jundishapur J. Nat. Pharm. Prod..

[27] Middleton P, Stewart F, Al-Qahtani S, Egan P, O’Rourke C, Abdulrahman A (2005). Antioxidant, antibacterial activities and general toxicity of Alnus glutinosa, Fraxinus excelsior and Papaver rhoeas. Iran. J. Pharm. Res..

[28] Adams RP (2001). Identification of essential oil of components by gas chromatography quadrupole mass spectroscopy.

[29] Peteros NP, Uy MM (2010). Antioxidant and cytotoxic activities and phytochemical screening of four Philippine medicinal plants. J. Med. Plant Res..

[30] Meyer B, Ferrigni N, Putnam J, Jacobsen L, Nichols Dj, McLaughlin J (1982). Brine shrimp: a convenient general bioassay for active plant constituents. Planta Medica..

[31] Kumar S, Kumar V, Chandrashekhar M (2011). Cytotoxic activity of isolated fractions from methanolic extract of Asystasia dalzelliana leaves by brine shrimp lethality bioassy. Int. J. Pharm. Pharm. Sci..

[32] Musa AA (2012). Cytotoxicity activity and phytochemical screening of Cochlospermum tinctorium Perr Ex A. rich rhizome. J. App. Pharm. Sci..

[33] Afshar FH, Delazar A, Nazemiyeh H, Esnaashari S, Moghadam SB (2012). Comparison of the total phenol, flavonoid contents and antioxidant activity of methanolic extracts of Artemisia spicigera and A splendens growing in Iran. Pharm. Sci..

[34] Saeed N, Khan MR, Shabbir M (2012). Antioxidant activity, total phenolic and total flavonoid contents of whole plant extracts Torilis leptophylla. BMC Comp. Alter Med..

[35] Delazar A, Byres M, Gibbons S, Kumarasamy Y, Modarresi M, Nahar L (2004). Iridoid glycosides from Eremostachys glabra. J. Nat. Prod..

[36] Fouladnia M, Modarresi M (2012). Phenylethanoid glycosides from Eremostachys azerbaijanica Rech. F. Res. Pharm. Sci..

[37] Modarresi M, Fouladnia M, Rafiee Z, jafari A, Zarzasangan K (2013). Iridoid glycosides from Eremostachys azerbaijanica Rech. F. root. J. Med. Plant..

[38] Anderson J, Goetz C, McLaughlin J, Suffness M (1991). A blind comparison of simple bench-top bioassays and human tumour cell cytotoxicities as antitumor prescreens. Phytochem. Analysis.

[39] Kiviranta J, Sivonen K, Niemelä S, Huovinen K (1991). Detection of toxicity of cyanobacteria by Artemia salina bioassay. Environ. Toxicol. Water Qual..

[40] Piccardi R, Frosini A, Tredici MR, Margheri MC (2000). Bioactivity in free-living and symbiotic cyanobacteria of the genus Nostoc. J. Appl. Phycol..

[41] Wu JT, Chiang YR, Huang WY, Jane WN (2006). Cytotoxic effects of free fatty acids on phytoplankton algae and cyanobacteria. Aquat. Toxicol..

[42] Igarashi M, Miyazawa T (2000). Newly recognized cytotoxic effect of conjugated trienoic fatty acids on cultured human tumor cells. Cancer Lett..

[43] Martins de Lima T, Cury-Boaventura M, Giannocco G, Nunes M, Curi R (2006). Comparative toxicity of fatty acids on a macrophage cell line (J774). Clinic Sci..

[44] Suzuki R, Noguchi R, Ota T, Abe M, Miyashita K, Kawada T (2001). Cytotoxic effect of conjugated trienoic fatty acids on mouse tumor and human monocytic leukemia cells. Lipids.

[45] Siegel I, Yaghoubzadeh E, Keskey TS, Gleicher N (1987). Cytotoxic effects of free fatty acids on ascites tumor cells. J. Nation Cancer Inst..

[46] Wu S-B, Bao Q-Y, Wang W-X, Zhao Y, Xia G, Zhao Z (2011). Cytotoxic triterpenoids and steroids. Planta Med..

[47] Kim KH, Choi SU, Kim CS, Lee KR (2012). Cytotoxic steroids from the trunk of Berberis koreana. Biosci. Biotech. Biochem..

[48] Ha TJ, Jang DS, Lee JR, Lee KD, Lee J, Hwang SW (2003). Cytotoxic effects of sesquiterpene lactones from the flowers of Hemisteptia lyrata B. Arch. Pharm. Res..

[49] Bai N, Lai CS, He K, Zhou Z, Zhang L, Quan Z (2006). Sesquiterpene lactones from Inula britannica and their cytotoxic and apoptotic effects on human cancer cell lines. J. Nat. Prod..

[50] Nord CL, Menkis A, Broberg A (2014). Cytotoxic illudalane sesquiterpenes from the wood-decay fungus Granulobasidium vellereum (Ellis & Cragin) Jülich. Molecules.

[51] Babich H, Zuckerbraun H, Weinerman S (2007). In-vitro cytotoxicity of catechin gallate, a minor polyphenol in green tea. Toxicol Lett..

[52] Durgo K, Vuković L, Rusak G, Osmak M, Franekić Čolić J (2009). Cytotoxic and apoptotic effect of structurally similar flavonoids on parental and drug-resistant cells of a human cervical carcinoma. Food Tech. Biotech..

[53] Mohareb RM, Al-Omran F, Azzam RA (2014). Heterocyclic ring extension of estrone: Synthesis and cytotoxicity of fused pyran, pyrimidine and thiazole derivatives. Steroids.

[54] Cheng JJ, Tsai TH, Lin LC (2012). New alkaloids and cytotoxic principles from sinomenium acutum. Planta Med..

[55] Kawase M, Sakagami H, Motohashi N, Hauer H, Chatterjee SS, Spengler G (2005). Coumarin derivatives with tumor-specific cytotoxicity and multidrug resistance reversal activity. In-vivo..

